# Draft Genome Sequences of *Candida glabrata* Isolates 1A, 1B, 2A, 2B, 3A, and 3B

**DOI:** 10.1128/genomeA.00328-16

**Published:** 2017-03-09

**Authors:** Othilde Elise Håvelsrud, Peter Gaustad

**Affiliations:** Department of Microbiology, Oslo University Hospital, Oslo, Norway

## Abstract

Here, we report the draft genome sequences of six *Candida glabrata* isolates. The isolates were taken from blood samples from patients after recurrent *C. glabrata* infection. Two isolates were taken from each of three patients a minimum 3 months apart.

## GENOME ANNOUNCEMENT

*Candida glabrata* is an important causal agent of candidemia in humans (1). A recent surveillance study from Norway, where our isolates were collected, identified *C. glabrata* as the most common causative agent of candidemia after *Candida albicans* (2). Here, we present the draft genome sequences of six isolates, two each from three patients with recurring candidemia. These draft genome sequences may help ascertain if the recurrent infections were caused by the same strain and whether the strain genomes evolve between infections. They also present a good opportunity for further investigation into the virulence of *C. glabrata*.

DNA was extracted using the YeaStar genomic DNA kit (Zymo Research), according to the manufacturer’s protocol I. The DNA was purified and concentrated using the Amicon Ultra-0.5 ml 30K centrifugal filters for DNA purification and concentration (Merck Millipore). The DNA concentration was determined by the Qubit double-stranded DNA (dsDNA) high-sensitivity (HS) assay kit (Life Technologies). The DNA quality was assessed by agarose gel electrophoresis and 260/280 ratios measured on a NanoDrop 2000 spectrophotometer (Thermo Scientific). Sequencing was performed at the Genomics Core Facility at Radiumhospitalet (http://oslo.genomics.no) using Illumina MiSeq technology. Approximately 15.4, 15.7, 16.3, 24.1, 15.6, and 14.2 million paired-end reads of 101 nucleotides (nt) were generated for *C. glabrata* isolates 1A, 1B, 2A, 2B, 3A, and 3B, respectively. After removal of read pairs containing ambiguous bases, the reads were assembled with Velvet version 1.2.10 and AMOScmp-shortReads version 3.1.0 (3, 4). VelvetOptimiser was used to set optimal parameter values for the Velvet assemblies. Only contigs >1,000 nt were retained in the Velvet assemblies. *C. glabrata* CBS138 (5, 6) was used as a reference for the AMOScmp-shortReads assemblies. Feature response curves were created in AMOS version 3.1.10 in order to evaluate assembly quality (4). Contigs from the two assemblies were joined with GAM-NGS version 1.1b using the AMOScmp-shortReads assembly as a master (7). SSPACE version 3.0 was used to scaffold the contigs (8). Both the intermediate contigs and the final scaffolds were validated with REAPR version 1.0.17 (9). mothur 1.36.1 was used to filter out all scaffolds <1,000 nt (10). The final assemblies consist of 12.6 Mbp (119 scaffolds), 12.6 Mbp (144 scaffolds), 14.6 Mbp (196 scaffolds), 12.8 Mbp (132 scaffolds), 12.8 Mbp (189 scaffolds), and 12.9 Mbp (183 scaffolds) for 1A, 1B, 2A, 2B, 3A, and 3B, respectively. The completeness of the assemblies was assessed with CEGMA version 2.4.010312 (11). The draft genome sequences were estimated to be 96 to 97% complete. The average G+C content was 38.6% for all draft genome sequences. The final scaffolds were annotated with MAKER version 2.31 (12). A total of 5,214 (1A), 5,191 (1B), 5,985 (2A), 5,293 (2B), 5,324 (3A), and 5,331 (3B) predicted protein-coding genes were detected. The predicted protein-coding genes cover approximately 65% of the draft genome sequences.

### Accession number(s).

These whole-genome shotgun projects have been deposited at DDBJ/EMBL/GenBank under the accession numbers LLWO00000000, LMAA00000000, LLZZ00000000, LLZY00000000, LMAY00000000, and LMAB00000000 for 1A, 1B, 2A, 2B, 3A, and 3B, respectively. The versions described in this paper are the first versions LLWO01000000, LMAA01000000, LLZZ01000000, LLZY01000000, LMAY01000000, and LMAB01000000, respectively.
